# Relationship between oral health and prognosis in patients with empyema: Single center retrospective study with propensity score matching analysis

**DOI:** 10.1371/journal.pone.0282191

**Published:** 2023-03-08

**Authors:** Eiji Iwata, Teruaki Nishiuma, Suya Hori, Keiko Sugiura, Masato Taki, Shuntaro Tokunaga, Junya Kusumoto, Takumi Hasegawa, Akira Tachibana, Masaya Akashi

**Affiliations:** 1 Department of Oral and Maxillofacial Surgery, Kakogawa Central City Hospital, Kakogawa, Japan; 2 Department of Respiratory Medicine, Kakogawa Central City Hospital, Kakogawa, Japan; 3 Department of Oral and Maxillofacial Surgery, Kobe University Graduate School of Medicine, Kobe, Japan; Jazan University Faculty of Dentistry, SAUDI ARABIA

## Abstract

**Background:**

Empyema is a life-threatening infection often caused by oral microbiota. To the best of our knowledge, no reports have investigated the association between the objective assessment of oral health and prognosis in patients with empyema.

**Materials and methods:**

A total of 63 patients with empyema who required hospitalization at a single institution were included in this retrospective study. We compared non-survivors and survivors to assess risk factors for death at three months, including the Renal, age, pus, infection, diet (RAPID) score, and Oral Health Assessment Tool (OHAT) score. Furthermore, to minimize the background bias of the OHAT high-score and low-score groups determined based on the cut-off value, we also analyzed the association between the OHAT score and death at 3 months using the propensity score matching method.

**Results:**

The 3-month mortality rate was 20.6% (13 patients). Multivariate analysis showed that a RAPID score ≥5 points (odds ratio (OR) 8.74) and an OHAT score ≥7 points (OR 13.91) were significantly associated with death at 3 months. In the propensity score analysis, a significant association was found between a high OHAT score (≥7 points) and death at 3 months (*P =* 0.019).

**Conclusion:**

Our results indicated that oral health assessed using the OHAT score may be a potential independent prognostic factor in patients with empyema. Similar to the RAPID score, the OHAT score may become an important indicator for the treatment of empyema.

## 1. Introduction

Empyema is defined as the presence of bacteria or pus in the pleural cavity and has common clinical symptoms, including dyspnea, fever, chest pain, and cough [1]. In recent years, the incidence of empyema has increased steadily worldwide, with a high mortality rate of 10–30% despite the advancements in antibiotic therapy and widespread chest tube drainage [2–4]. Physicians must understand the risk factors, clinical features, and severity of diseases with high mortality. A clinical risk score for predicting death can facilitate the formulation of early management strategies. In 2014, the RAPID score was developed as a clinical risk score of pleural infections, including empyema [5]. This score is composed of the following five patient characteristics and clinical data: renal, age, purulence, infection source, and dietary factors. Recent reports have revealed an association between the RAPID score and 3-month death rate (low risk: 0–2 points, medium risk: 3–4 points, high risk: 5–7 points) in patients with empyema [6,7].

The most common cause of empyema is bacterial pneumonia, which is associated with oral health, including the number of oral bacteria [8,9]. Bacteria breach the visceral pleura to establish an infected parapneumonic effusion, resulting in empyema. Many studies have reported that oral bacteria, including *Streptococcus spp*., *Staphylococcus aureus*, and *Fusobacterium spp*. Have been detected as the main causative bacteria of empyema [10–12]. In addition, a recent report presented direct genetic evidence that some bacteria in empyema are derived from the oral flora [13]. Therefore, oral health may be associated with the onset or prognosis of empyema. To the best of our knowledge, no reports have investigated the association between the objective assessment of oral health and prognosis in patients with empyema. The Oral Health Assessment Tool (OHAT) score system is widely recognized as an objective tool for assessing oral health [14–17]. In 2015, this scoring system was developed for non-dental professionals such as nurses and allied health personal care attendants [14]. This score consists of the following eight categories on a 3-point scale: lips, tongue, gums and mucosa, saliva, natural teeth, dentures, oral cleanliness, and dental pain, with higher total scores indicating poorer overall oral health. Recently, many researchers, including dentists, have used the OHAT score to evaluate the oral health of patients in various fields [15–17]. This study aimed to investigate the association between oral health assessed using the OHAT score and prognosis in patients with empyema.

## 2. Patients and methods

### 2.1. Patients

This study included 63 patients hospitalized for empyema treatment between January 2017 and July 2022 at Kakogawa Central City Hospital. Light’s classification was used to diagnose empyema [18]. In brief, 1) aspiration of grossly purulent material on thoracentesis and 2) at least one of the following: a) thoracentesis fluid with a positive Gram stain or culture, b) pleural fluid glucose <40 mg/dL, c) pH <7.2, or d)- lactate dehydrogenase >1000 IU/L [18]. The exclusion criteria were as follows: patients under 20 years old, those who did not undergo pleural puncture for some reason, those who did not wish to participate after the publication of this study, and those with missing data that were needed in this study. Patients with confirmed empyema underwent various tests such as blood tests, and were treated with antibiotics and chest tube drainage. They also underwent dental examinations, including panoramic dental radiography and oral photography, within days after hospitalization and dental treatments, if needed, during hospitalization.

This study was performed in accordance with the 1964 Declaration of Helsinki. Ethical approval was obtained from the Institutional Review Boards (IRB) of Kakogawa Central City Hospital (Authorization number: 2020–46). The ethics committee approved the study and gave administrative permissions to access the data used in this study. As this was a retrospective study, the research plan was published on the homepage of the hospital according to the instructions of the IRB in accordance with the guaranteed opt-out opportunity.

### 2.2. Study design

The present study is a retrospective cohort study. Patients were divided into two groups: non-survivors and survivors at 3 months. The following variables from medical records were investigated: (1) patient factors (sex, presence of dysphagia, compromised-host, smoking history); (2) clinical findings factors, such as CRP, WBC, blood urea nitrogen (BUN), age, purulence of pleural fluid, infection source (community-acquired/hospital-acquired), serum albumin, OHAT score, and etiology (monomicrobial/polymicrobial/no growth); and (3) treatment methods. Dysphagia was defined as coughing when taking a meal or decreasing swallowing ability on evaluation by physicians and speech-language-hearing therapists [7]. Data on treatment and outcomes were also evaluated for each patient during hospitalization. A compromised-host was defined as a patient with any of the following diseases: rheumatoid arthritis, chronic kidney disease, malignancy, diabetes, cardiovascular diseases, neurological diseases, and steroid use. We used two clinical risk scores: RAPID (total score; min:0 point, max:7 points) and OHAT (total score; min:0 point, max:16 points). The RAPID score was based on five common parameters (Table 1) [6]. Based on the results of the dental examinations, the presence of teeth with poor prognosis was retrospectively investigated using panoramic dental radiography. They were defined as teeth with abnormal radiographic findings (e.g., apical radiolucency larger than 3 mm in diameter, alveolar bone loss around more than half of the root, untreated root remnants, or vertically fractured roots) [19,20]. Medical records were used whether those teeth were extracted. Pleural fluid was collected by pleural puncture at the time of admission, and microbiological examinations were performed. Anaerobic containers were used to collect pleural fluid to detect anaerobic bacteria, and Gram staining and pleural fluid cultures were performed. Blood agar (Kohjin Bio Co., Ltd., Saitama, Japan) and chocolate agar media (Kohjin Bio Co., Ltd.) were used to detect general bacteria. Anaero Columbia agar medium with hemin and vitamin K1 (Nippon Becton Dickinson Co., Ltd., Tokyo, Japan) was used to detect anaerobic bacteria; any anaerobic bacteria were then cultivated at 35°C and 9% CO_2_. The causative pathogens were then identified in the pleural fluid culture.

**Table 1:** RAPID score.

RAPID score		
Parameter	Measure	Score
Renal BUN (mg/dL)	<14	0
Age (years) Purulence of pleural fluid Infection source Dietary factors Alb (g/dL) Risk categories	14–22.4 >22.4 <50 >70 Purulent Non-purulent Community-acquired Hospital-acquired ≥2.7 <2.7 Score 0–2 Score 3–4 Score 5–7	1 2 0 1 2 0 1 0 1 0 1 Low risk Medium risk High risk

### 2.3. OHAT score

The OHAT score consists of eight categories with three possible scores (0 = healthy, 1 = some changes, and 2 = unhealthy) (Table 2) [14]. The total score is the sum of the various sub-scores. Based on the results of the dental examinations, including oral photographs and medical records, OHAT score of each patient was retrospectively evaluated by two observers (EI and KS). EI is an oral and maxillofacial surgeon with ≥ 10 years of experience, and KS is a dental hygienist with ≥ 10 years of experience. The OHAT-J, which includes images of each category and point scale in Japanese, is well-known among dentists and dental hygienists in Japan [21,22]. In this study, the dentist (EI) and dental hygienist (KS) evaluated the OHAT score after visual training and calibration by using this picture (S1 Data). Finally, the OHAT score of each patient was determined through discussion among the observers.

**Table 2:** OHAT score.

OHAT score			
Category	0 = healthy	1 = changes	2 = unhealthy
Lips	Smooth, pink, moist	Dry, chapped, or red, at corners	Swelling or lump, white/red/ulcerated patch; bleeding/ulcerated at corners
Tongue Gums and tissues Saliva Natural teeth Dentures Oral cleanliness Dental pain	Normal, moist, rough, pink Pink, moist, smooth, no bleeding Moist tissues, watery and free flowing saliva No decayed or broken teeth/roots No broken areas or teeth, dentures regularly worn and named Clean and no food Particles or tartar in mouth or dentures No behavioral, verbal, or physical signs of dental pain	Patchy, fissured, red, coated Dry, shiny, rough, red, swollen, one ulcer/sore spot under dentures Dry sticky tissues, little saliva present, resident thinks they have a dry mouth 1–3 decayed or broken teeth/roots or very worn-down teeth 1 broken area/tooth or dentures only worn for 1–2 h daily, or dentures not named or loose Food particles/tartar/plaque in 1–2 areas of the mouth or on a small area of dentures or halitosis (bad breath) Verbal and/or behavioral signs of pain present, such as pulling at face, chewing lips, not eating, aggression	White/red patches, ulcerated, swollen Swollen, bleeding ulcers, white/red patches, generalized redness under dentures Tissue parched and red, very little/no saliva present, saliva thick, resident thinks they have a dry mouth ≥4 decayed or broken teeth/roots, or very worn-down teeth, or <4 teeth More than 1 broken area/tooth, denture missing or not worn, loose and needs denture adhesive, or not named Food particles/tartar/plaque in most areas of the mouth or on most areas of dentures or severe halitosis (bad breath) Physical pain signs (swelling of cheek or gum, broken, ulcers) present, as well as verbal and/or behavioral signs (pulling at face, not eating, aggression)

### 2.4. Statistical analyses

Statistical analyses were performed using SPSS (version 26.0; IBM Corp., Armonk, NY, USA) and Ekuseru-Toukei 2012 (Social Survey Research Information Co., Ltd., Tokyo, Japan). A receiver operating characteristic (ROC) curve was used to determine the cut-off values for the RAPID and OHAT scores. The area under the ROC curve was used to measure the accuracy of discrimination. The area under the ROC curve was used to measure the accuracy of discrimination (range, 0.5 to 1). The association of each variable with death at 3 months was analyzed using the non-parametric Mann-Whitney U test for ordinal variables and either the Fisher’s exact test or the chi-squared test was used for categorical variables. Statistical significance was set at *P*<0.05. The selected variables were included in a multiple logistic regression model using the forced-entry method. Odds ratios (Ors) and 95% confidence intervals (Cis) were calculated. Furthermore, to minimize selection bias associated with the comparison of retrospective data analysis, propensity score matching was performed between the high and low OHAT score groups using cut-off values. Subsequently, propensity score-matched cases (36) were evaluated to determine an association between a high OHAT score and death at 3 months. Reliability assessments for the stability of OHAT scores were assessed in a test-retest of observers using Cohen’s kappa statistic for the individual categories and intraclass correlation (ICC) for the total OHAT score [14]. The Kappa statistic indicated the degree of departure between the actual observed and chance percentage agreement and was not weighted. In interpreting the Kappa statistic, values of 0.41–0.60 were considered moderate, 0.61–0.80 substantial, and 0.81–1.0 almost perfect agreement [14].

## 3. Results

The 3-month mortality rate was 20.6% (13 out of 63 patients). The median age of non-survivors was 84.0 years and that of survivors was 72.0 years, which showed a significant difference (*P*<0.001). All non-survivors and 42 of the 50 survivors were male.

Table 3 shows patient characteristics and the results of the univariate analysis. In the univariate analysis, the rate of dysphagia (*P = 0*.*047*), RAPID score (*P<0*.*001*), and OHAT score (*P<0*.*001*) were significantly higher in non-survivors than in survivors. Of the five factors assessed by the RAPID score, BUN level, and age were significantly higher in non-survivors than in survivors, while serum albumin level was lower in non-survivors than in survivors. Of the eight categories of OHAT score, lip (*P*<0.001), tongue (*P* = 0.021), gums and tissues (*P* = 0.007), and saliva (*P* = 0.014) were unhealthier in non-survivors than survivors. More than half of the non-survivors (69.2%) and survivors (54.0%) had teeth with a poor prognosis. Of them, some non-survivors (22.2%) and survivors (55.6%) underwent extraction of those teeth. Non-survivors tended to have a lower frequency of oral care than survivors (*P* = 0.135).

**Table 3:** Distribution of variables between non-survivors and survivors.

Variables		Non-survivors Survivors (n = 13) (n = 50)		*P* value
**Sex**	Male	13 (100.0%)	42 (84.0%)	0.188 ^b^
**Dysphagia**	Yes	12 (92.3%)	31 (62.0%)	0.047* ^**b**^
**Compromised host**	Yes	6 (46.2%)	19 (38.0%)	0.752 ^b^
**Smoking history**	Yes	10 (76.9%)	47 (74. 0%)	1.000 ^b^
**CRP (mg/dL)**	Median (range)	23.7 (7.1–30.3)	21.4 (2.1–41.0)	0.425 ^a^
**WBC (10** ^**3**^ **/μL)**	Median (range)	15.6 (10.8–24.8)	15.4 (5.5–39.2)	0.663 ^a^
**RAPID score**	Median (range)	5.0 (2–6)	3.0 (0–5)	<0.001* ^a^
BUN (mg/dL) Age (years)	Median (range) Median (range)	43.4 (9.4–95.4) 84.0 (65–96)	16.3 (4.6–67.5) 72.0 (43–90)	<0.001* ^a^ <0.001* ^a^
Purulence of pleural fluid	Purulent Non-purulent	13 (100.0%) 0 (0.0%)	42 (87.3%) 8 (12.7%)	0.188 ^b^
Infection source	Community-acquired	12 (92.3%)	45 (90.0%)	1.000 ^b^
	Hospital-acquired	1 (7.7%)	5 (10.0%)	
Serum albumin (g/dL)	Median (range)	2.1 (1.2–2.9)	2.5 (1.7–4.2)	0.025* ^a^
**OHAT score**	Median (range)	7.0 (4–13)	5.0 (0–11)	<0.001* ^a^
Lips	Median (range)	1.0 (0–2)	0.0 (0–2)	<0.001* ^a^
Tongue	Median (range)	1.0 (0–2)	1.0 (0–2)	0.021* ^a^
Gums and tissues	Median (range)	1.0 (0–2)	0.5 (0–2)	0.007* ^a^
Saliva Natural teeth	Median (range) Median (range)	1.0 (0–2) 1.0 (0–2)	0.0 (0–2) 1.0 (0–2)	0.014* ^a^ 0.697 ^a^
Teeth with a poor prognosis	Presence	9 (69.2%)	27 (54.0%)	0.365 ^b^
With tooth extraction	Yes (/presence)	2/9 (22.2%)	15/27 (55.6%)	0.128 ^b^
Dentures Oral cleanliness Frequency of oral care Dental pain	Median (range) Median (range) Median (range) Median (range)	0.0 (0–2) 2.0 (0–2) 2.0 (0–3) 0.0 (0)	0.0 (0–2) 2.0 (0–2) 2.0 (1–4) 0.0 (0–2)	0.913 ^a^ 0.055 ^a^ 0.135 ^a^ 0.384 ^a^
**Etiology**	Monomicrobial	5 (38.5%)	22 (44.0%)	1.000 ^b^
	Polymicrobial	2 (15.3%)	11 (22.0%)	
	No growth	6 (46.2%)	17 (34.0%)	
**Treatment**	Antibiotic therapy only	4 (30.8%)	4 (8.0%)	0.120 ^c^
	+ drainage	2 (15.4%)	5 (10.0%)	
	+ drainage, urokinase	7 (53.8%)	39 (78.0%)	
	+ surgery	0 (0.0%)	2 (4.0%)	

Values are expressed as absolute numbers, with the corresponding percentage of the total in parentheses. Values in the right-hand column indicate the statistical significance of the difference between subgroups. Most variables expressed as the median (range) in a non-parametric ratio scale.

^a^Mann-Whiteny U test;

^b^Fisher’s exact test;

^c^Chi-squared test.

*P < 0.05.

**Fig 1:**
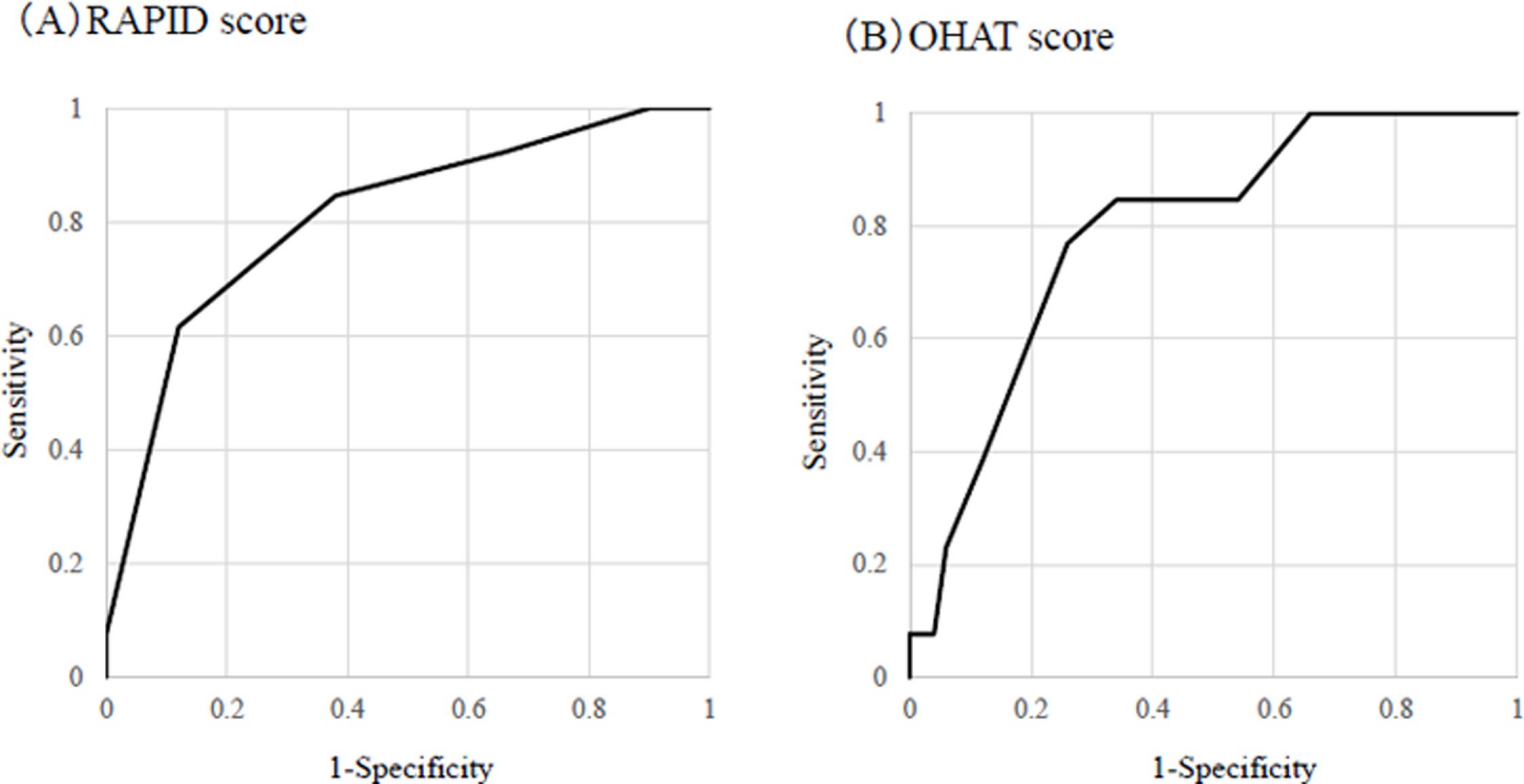
(A) The ROC curve for accuracy of RAPID score. The AUC for our model was 0.810 (95% confidence interval 0.675 to 0.945). (B) The ROC curve for accuracy of OHAT score. The AUC for our model was 0.790 (95% confidence interval 0.664 to 0.91.

RAPID score ≥5 points had a sensitivity of 61.5%, a specificity of 88.0%, and an area under curve (AUC) of 0.81 (Fig 1A). OHAT score ≥7 points had a sensitivity of 76.9%, a specificity of 74.0%, and an AUC of 0.79 (Fig 1B). After applying a logistic regression model and forced entry method, we found that RAPID score ≥5 points (OR 8.74) and an OHAT score ≥7 points (OR 13.91) were significant risk factors for death at 3 months (Table 4). Table 5 includes the intra- and inter-rater reliability data for the OHAT scores. Intra-rater reliability ranged from 84.1% for oral cleanliness to 100% for dental pain. Kappa statistics were in the range considered substantially perfect (0.61–0.80) for saliva and oral cleanliness, and for all other categories in the range of 0.81–1.00 (almost perfect). Inter-rater reliability ranged from 82.5% for oral cleanliness to 100% for dental pain. Kappa statistics were in the range of substantially perfect (0.61–0.80) for lips, saliva, and oral cleanliness, and for all other categories in the range of 0.81–1.00 (almost perfect). The ICC for the OHAT total scores was 0.94 for intra-rater and 0.92 for inter-rater reliability.

**Table 4:** The results of the multivariate logistic regression analysis of the risk factors for death at three months in empyema.

			95% CI	
Variable	*P* value	Odds ratio	Lower	Upper
Sex Male	.999	390596307.6	.000	
Dysphagia	.247	4.909	.333	72.429
Compromised host	.888	.877	.142	5.413
Smoking history High CRP (21.8≥) High WBC (14170≥) High RAPID score (≥5 points)	.409 .064 .128 .019*	2.693 8.672 .192 8.742	.256 .881 .023 1.432	28.314 85.365 1.611 53.377
High OHAT score (≥7 points)	.013*	13.905	1.755	110.160
CI. Confidence interval				

Data are the p-value, OR and 95% CI for those factors found to be significantly associated with death at three months in empyema.

*P < 0.05.

**Table 5:** Intra-rater and inter-rater reliability for individual OHAT categories and total OHAT score.

Category	Intra-rater		Inter-rater	
	Percent agreement	Kappa statistic	Percent agreement	Kappa statistic
Lips	90.5	0.83*	87.3	0.77*
Tongue	90.5	0.84*	88.9	0.81*
Gums and tissues	92.1	0.88*	90.5	0.83*
Saliva	88.9	0.79*	87.3	0.76*
Natural teeth	93.7	0.90*	90.5	0.86*
Dentures	96.8	0.89*	95.2	0.84*
Oral cleanliness	84.1	0.76*	82.5	0.73*
Dental pain	100.0	1.00*	100.0	1.00*
	Intraclass correlation		Intraclass correlation	
Total OHAT score	0.94*		0.92*	

*P < 0.001.

Table 6 shows the results of the pleural fluid culture test. In both groups, oral bacteria were detected in many patients. The most frequently detected bacteria were *Streptococcus species*, followed by *facultative* anaerobic *Staphylococcus* spp., obligate anaerobic *Prevotella* spp., *Parvimonas micra*, and *Porphyromonas gingivalis*.

**Table 6:** Distribution of microorganisms as the cause of empyema.

Non-survivors	No.	Survivors	No.
[Facultative anaerobic bacteria]	5	[Facultative anaerobic bacteria]	31
*Streptococcus* spp.	3	*Streptococcus* spp.	22
*S*. *constellatus*	(2)	*S*. *intermedius*	(10)
*S*. *intermedius*	(1)	*S*. *anginosus*	(4)
*Psudomonas aeruginosa*	1	*S*. *constellatus*	(4)
*Aspergillus fumigatus*	1	*S*. *agalactiae*	(2)
		*S*. *gordonii*	(1)
		*S*. *mitis*	(1)
		*Staphylococcus* spp.	5
		*S*. *aureus*	(2)
		MRSA	(2)
		MSSA	(1)
		*Psudomonas aeruginosa*	2
		*Citrobacter koseri*	1
		*Enterococcus faecalis*	1
[Obligate anaerobic bacteria]	5	[Obligate anaerobic bacteria]	13
*Prevotella* spp.	2	*Parvimonas micra*	3
*P*. *buccae*	(1)	*Prevotella* spp.	3
*P*. *disiens*	(1)	*P*. *buccae*	(1)
*Parvimonas micra*	1	*P*. *disiens*	(1)
*Porphyromonas gingivalis*	1	*P*. *denticola*	(1)
*Fusobacterium nucleatum*	1	*Porphyromonas gingivalis*	3
		*Veillonella* spp.	1
		*Bacteroides vulgatus*	1
		*Fusobacterium nucleatum*	1
		*Finegoldia magna*	1

We compared patient characteristics between the OHAT high-score (≥ 7 points) and low-score (< 7 points) groups (Table 7). Propensity score matching was performed for an unbiased analysis of the OHAT score using seven variables (sex, dysphagia, compromised host, smoking history, CRP, WBC, and RAPID score). After propensity score matching, the characteristics of the two groups were balanced in seven variables, and the rate of non-survivors was significantly higher in the OHAT high-score group than in the low-score group (*P =* 0.019) (Table 8).

**Table 7:** Background factors of patients with high and low OHAT score.

Variables		High OHAT score Low OHAT score (n = 23) (n = 40)		*P* value
Sex	Male	18 (78.3%)	37 (92.5%)	0.129 ^b^
Dysphagia	Yes	17 (73.9%)	26 (65.0%)	0.578 ^b^
Compromised host	Yes	8 (34. 8%)	17 (42.5%)	0.602 ^b^
Smoking history	Yes	15 (65.2%)	32 (80.0%)	0.236 ^b^
CRP (mg/dL)	Median (range)	23.7 (7.1–40.5)	21.0 (2.1–41.0)	0.589 ^a^
WBC (10^3^/μL)	Median (range)	16.5 (10.7–39.2)	13.7 (5.5–28.7)	0.139 ^a^
RAPID score	Median (range)	4.0 (2–6)	3.0 (0–5)	0.006* ^a^

Values are expressed as absolute numbers, with the corresponding percentage of the total in parentheses. Values in the right-hand column indicate the statistical significance of the difference between subgroups. Most variables expressed as the median (range) in a non-parametric ratio scale.

^a^Mann-Whiteny U test;

^b^Fisher’s exact test.

*P < 0.05.

**Table 8:** Background factors of patients with high and low OHAT score after propensity score matching.

Variables		High OHAT score Low OHAT score (n = 18) (n = 18)		*P* value
Sex	Male	13 (72.2%)	16 (88.9%)	0.402 ^b^
Dysphagia	Yes	12 (66.7%)	11 (61.1%)	1.000 ^b^
Compromised host	Yes	7 (38.9%)	6 (33.3%)	1.000 ^b^
Smoking history	Yes	13 (72.2%)	13 (72.2%)	1.000 ^b^
CRP (mg/dL)	Median (range)	22.7 (7.1–40.0)	21.8 (2.1–31.1)	0.877 ^a^
WBC (10^3^/μL)	Median (range)	15.2 (10.7–39.2)	15.6 (7.9–28.7)	0.570 ^a^
RAPID score Outcome	Median (range) Non-survivors	4.0 (2–5) 6 (33.3%)	3.0 (0–5) 0 (0.0%)	0.273 ^a^ 0.019* ^b^

Values are expressed as absolute numbers, with the corresponding percentage of the total in parentheses. Values in the right-hand column indicate the statistical significance of the difference between subgroups. Most variables expressed as the median (range) in a non-parametric ratio scale.

^a^Mann-Whiteny U test;

^b^Fisher’s exact test.

*P < 0.05.

## 4. Discussion

In this study, we investigated the risk factors for death at 3 months in patients with empyema. Multivariate analyses showed that a RAPID score ≥5 points (OR 8.74) and an OHAT score ≥7 points (OR 13.91) were significantly associated with death at 3 months. Additionally, using cut-off values, propensity score analysis between the high and low OHAT score groups revealed a significant association between OHAT high score (≥7 points) and death at 3 months (*P =* 0.019).

The 3-month mortality rate in this study was 20.6%, which was slightly higher than or similar to that reported in previous studies [2–5,7]. In addition to the RAPID and OHAT scores, univariate analysis showed that age (*P<0*.*001*) out of RAPID score and presence of dysphagia (*P = 0*.*047*) were significantly associated with death at 3 months in patients with empyema. Pneumonia is the third leading cause of death in Japan (9.2%), and the ratio of aspiration pneumonia to total cases of pneumonia increases with age (50–59 years: approximately 30%; 60–69 years: approximately 50%; 70–79 years: approximately 70%; 80–89 years: approximately 80%; over 90 years: approximately 90%) [23]. In general, 20–40% of hospitalized patients with pneumonia have pleural effusion, and 10% progress to acute empyema [18]. A previous study reported a significant relationship between dysphagia and death at 3 months in patients with empyema [13].

Previous studies reported that the RAPID score enables the prediction of death in patients with pleural infections, including empyema at 3 months [5–7], indicating that patients with RAPID scores ≥5 were at a high risk of death at 3 months [6,7]. These results are in line with those of the present study. Of the five factors contributing to this score, BUN levels, age, and serum albumin levels were significantly different between survivors and non-survivors. High BUN levels indicate dehydration, which is expected to negatively affect the patient prognosis. A previous report showed that high BUN levels in the RAPID score were associated with death at 3 months in patients with empyema (median 53 mg/dL vs 19 mg/dL; *P*<0.01) [7] similarly to this study (median 43.4 mg/dL vs 16.3 mg/dL; *P*<0.001). Serum albumin is a reliable marker of nutritional status [24], and a previous study showed an association between low serum albumin levels and infection [25]. Additionally, Sakai et al. reported that preoperative serum albumin level is a valid predictor of complications following surgery for acute empyema (incidence of high-level group vs low-level group = 39% vs 8%; *P* = 0.012) [26].

The OHAT score has been used in various fields [15–17], and its inter-rater reliability has been discussed. Several researchers have used Cohen’s kappa statistics to investigate the reliability and validity of the OHAT score per category in many fields [14,27,28], concluding that the OHAT score is a reliable and valid screening assessment tool for their research subjects. We also analyzed the intra- and inter-rater reliabilities of the OHAT scores per category using Cohen’s kappa statistics in this study. The results were either almost perfect or moderately perfect and were reliable for evaluation. Furthermore, Nishizawa et al. investigated the association between the OHAT score and aspiration pneumonia [29]. They set the OHAT score cut-off value to 4 points, and few patients with aspiration pneumonia had OHAT scores of ≥7 points, which was the cut-off value determined by using ROC curve in the present study. This difference may indicate the patient’s general medical condition during each disease.

Empyema is caused by obligate anaerobic bacteria such as *Prevotella* spp., *Peptostreptococcus*, or *Fusobacterium nucleatum* (30–40% responsible for mixed infections) in addition to *Streptococcus pneumoniae* and *Staphylococcus aureus* [10–12,30,31]. The detection frequency of *the Streptococcus anginosus* group (*S*. *anginosus*, *S*. *constellatus*, and S. *intermedius*), which resides in the oral cavity, is also high. Particularly in empyema, polymicrobial infections with obligate anaerobic bacteria are common [32]. In the present study, *Streptococcus pneumoniae* was not detected, and there was no significant difference between monomicrobial and polymicrobial patients, unlike the results of a previous study in which a significant difference was found [13]. The most frequently detected bacteria were *Streptococcus anginosus*, followed by *Staphylococcus* spp., *Prevotella* spp., *Parvimonas micra*, and *Porphyromonas gingivalis*. Therefore, most of the causative bacteria were derived from the oral flora. Teeth and periodontal tissues can be a route of bacterial invasion [33]. There are two possible pathways for the onset of empyema: first, descending mediastinitis by dental infection (via the cervical tissue space) spreading into the thoracic cavity; and second, the route by which bacteria reach the thoracic cavity via hematogenous circulation [33]. In this study, two out of nine non-survivors with teeth with a poor prognosis (22.2%) and 15 of 27 survivors (55.6%) underwent tooth extraction. Non-survivors tended to have a lower frequency of oral care than survivors (*P* = 0.135). Many non-survivors did not wish to improve their oral health by extracting teeth with a poor prognosis or frequent oral care, regardless of their higher OHAT score than survivors. Therefore, our dental education, might have been insufficient, especially for the OHAT high-score groups. Patients with a high OHAT score and who leave teeth with a poor prognosis untreated may have a low interest in oral health. Dentists should educate patients on the importance of improving oral health to improve the prognosis of empyema.

This study had several limitations. First, there is a possibility of unknown confounding factors as this was a retrospective study; for example, degree of underlying disease (e.g., presence of chronic obstructive pulmonary disease) or degree of smoking history (e.g., Brinkman index). Although, a propensity score matching analysis was performed to decrease the effect of confounding factors as much as possible, the possibility of selection bias could not be completely excluded. Second, the sample size was small, which might have introduced biases in the data selection and analyses. Third, no bacteria were detected in the pleural fluid cultures of several patients. One possible reason is that some patients may have undergone pleural puncture after antibiotic administration. Whether antibiotics were administered before hospitalization is unknown. However, strict limitations of antibiotic administration in clinics or other hospitals before admission to our hospital may be difficult because empyema is a severe disease that can result in death, and early management is important. Additionally, we did not use quantitative polymerase chain reaction or next-generation sequencing to detect the causative bacteria. Finally, the content of dental treatment may have affected the prognosis of patients with empyema. In this study, the dental treatment methods, including the standard of tooth extraction and frequency of oral care, were not unified. However, there was no significant difference between non-survivors and survivors in the rate of patients with tooth extraction and frequency of oral care. In the future, prospective studies are necessary to identify useful prognostic factors for patients with empyema.

## 5. Conclusion

This is the first report to investigate the association between the objective assessment of oral health and the prognosis of empyema. Our results indicate that oral health assessed using the OHAT score may be a potential independent prognostic factor in patients with empyema. However, these findings should be carefully considered because of the retrospective study design. In a prospective study, we should eliminate confounding factors as much as possible by excluding patients with the administration of antibiotics before pleural puncture and unifying dental treatment methods, such as standard of tooth extraction and frequency of oral care.

## Supporting information

S1 Data OHAT-J. Quote source: Website of Department of Oral Health Sciences for Community Welfare, Tokyo Medical and Dental University Graduate School, Tokyo, Japan http://www.ohcw-tmd.com/research/ohat.html.
(PDF)
