# Supplementary material for: Relationship between oral health and prognosis in patients with empyema: Single center retrospective study with propensity score matching analysis
Source: PLoS One. 2023 Mar 8;18(3):e0282191. doi: 10.1371/journal.pone.0282191 (PMC9994691; doi:10.1371/journal.pone.0282191)
Supplement: S1 Data — Quote source: Website of Department of Oral Health Sciences for Community Welfare, Tokyo Medical and Dental University Graduate School, Tokyo, Japan http://www.ohcw-tmd.com/research/ohat.html. (PDF) [file pone.0282191.s001.pdf]

ORAL HEALTH ASSESSMENT TOOL 日本語版（OHAT-J）

(Chalmers JM, 2005; 松尾, 2016)

ID:	氏名：			評価日：　　/　　/			
項　目		0＝健　全		1＝やや不良		2＝病　的	スコア
口　唇	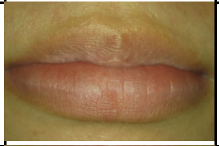	正常，湿潤，ピンク	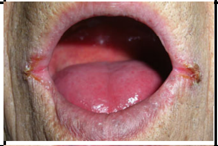	乾燥，ひび割れ，口角の発赤	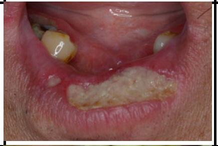	腫脹や腫瘤，<br>赤色斑，白色斑，潰瘍性出血，<br>口角からの出血，潰瘍	
舌	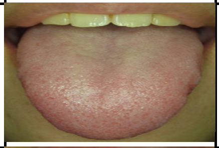	正常，湿潤，ピンク	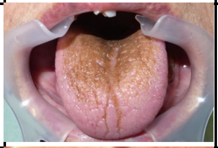	不整，亀裂，発赤，舌苔付着	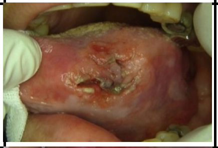	赤色斑，白色斑，潰瘍，腫脹	
歯肉・粘膜	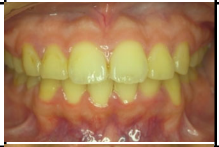	正常，湿潤，ピンク	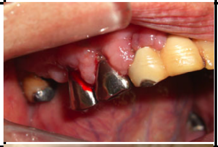	乾燥，光沢，粗造，発赤<br>部分的な(1-6歯分)腫脹<br>義歯下の一部潰瘍	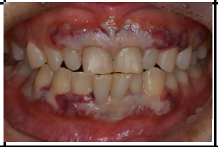	腫脹，出血(7歯分以上)<br>歯の動揺，潰瘍<br>白色斑，発赤，圧痛	
唾　液	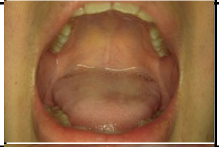	湿潤，漿液性	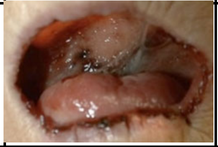	乾燥，べたつく粘膜，<br>少量の唾液<br>口渇感若干あり	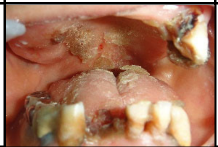	赤く干からびた状態<br>唾液はほぼなし，粘性の高い唾液<br>口渇感あり	
残存歯<br>□有　□無	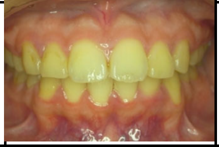	歯・歯根の<br>う蝕または破折なし	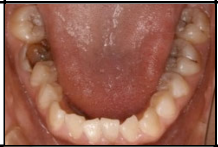	3本以下の<br>う蝕，歯の破折，残根，咬耗	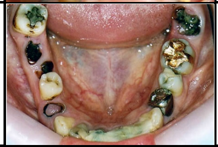	4本以上のう蝕，歯の破折，残根<br>非常に強い咬耗<br>義歯使用無しで3本以下の残存歯	
義　歯<br>□有　□無	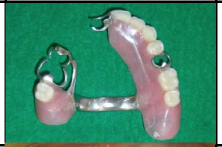	正常<br>義歯，人工歯の破折なし<br>普通に装着できる状態	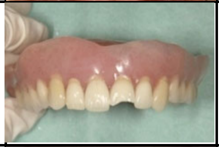	一部位の義歯，人工歯の破折<br>毎日1-2時間の装着のみ可能	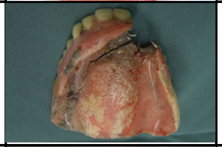	二部位以上の義歯，人工歯の破折<br>義歯紛失，義歯不適のため未装着<br>義歯接着剤が必要	
口腔清掃	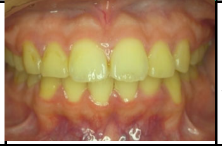	口腔清掃状態良好<br>食渣，歯石，プラークなし	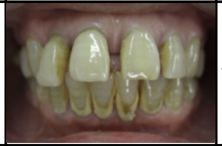	1-2部位に<br>食渣，歯石，プラークあり<br>若干口臭あり	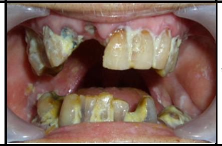	多くの部位に<br>食渣，歯石，プラークあり<br>強い口臭あり	
歯　痛	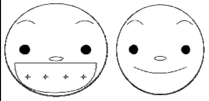<br>0　1	疼痛を示す<br>言動的，身体的な兆候なし	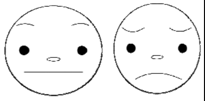<br>2　3	疼痛を示す言動的な兆候あり：<br>顔を引きつらせる、口唇を噛む<br>食事しない、攻撃的になる	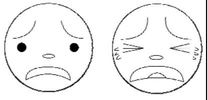<br>4	疼痛を示す身体的な兆候あり：<br>頬、歯肉の腫脹、歯の破折、潰瘍<br>歯肉下膿瘍。言動的な徴候もあり	
歯科受診　（　要　・　不要　）			再評価予定日　　/　　/			合計	

Japanese Translation: Koichiro Matsuo permitted by The Iowa Geriatric Education Center

avairable for download: https://www.ohcw-tmd.com/research/　　*revised Sept 1, 2021*

日本語版作成：東京医科歯科大学大学院地域・福祉口腔機能管理学分野　教授　松尾　浩一郎
